# The Influence of Tree Characteristics on White‐Backed Vulture (*Gyps africanus*) Nest‐Site Selection in Manyeleti and Kempiana Nature Reserves, South Africa

**DOI:** 10.1002/ece3.72545

**Published:** 2025-11-17

**Authors:** Stanislas Mahussi Gandaho, Ezéchiel Fidèle Koffi Hounnouvi, Lindy Jane Thompson, Fern Bain, Paul Scholte, Peter Hamming

**Affiliations:** ^1^ Laboratory of Applied Ecology University of Abomey‐Calavi Cotonou Benin; ^2^ ERAIFT, University of Kinshasa Kinshasa Republic of the Congo; ^3^ Centre for Functional Biodiversity, School of Life Science University of KwaZulu‐Natal Pietermaritzburg South Africa; ^4^ Southern African Wildlife College, Springvalley Farm 200KU Hoedspruit South Africa; ^5^ GIZ Addis Ababa Ethiopia

**Keywords:** *Gyps africanus*, Manyeleti and Kempiana Nature Reserves, nest‐site selection, potential nesting tree, tree health, tree size, vulture habitat, White‐backed vulture

## Abstract

An understanding of nesting tree characteristics is essential for the conservation of Critically Endangered White‐backed Vultures ( *Gyps africanus* ). To assess the influence of tree characteristics and tree health on White‐backed Vulture nest‐site selection, we measured a total of 205 trees (including vulture nest trees and randomly chosen trees) in Manyeleti and Kempiana Nature Reserves, South Africa. For each tree, we recorded trunk circumference at 1.3 m (CBH1) and 0.3 m (CBH2) above ground level, canopy width at its widest point, and total height. Tree health was evaluated based on trunk damage, including fire scars, insect infestation, fungal presence, and elephant debarking. *Diospyros mespiliformis* was the most frequently selected tree species (10.2% active nests). Most trees (74.2%) were healthy, 18.1% were unhealthy, and 7.8% were very unhealthy, primarily due to debarking by African savanna elephants ( *Loxodonta africana* ). There was no significant relationship between tree health status and vulture nest presence. Vultures prioritized tree size over tree health. Logistic regression identified trunk circumference as a key predictor (*p* = 0.030, *z* value = 2.175), with larger trees associated with increased odds of nesting (coefficient: 0.587 ± 0.270), likely due to better support for the nests and higher elevation. However, insects and fungi reduced tree survival, and this was worsened by elephant damage. Conservation practitioners should focus on protecting *Diospyros mespiliformis* by managing elephant densities and controlling burns to mitigate vulture nesting tree damage and habitat stress.

## Graphical abstract

White‐backed Vultures (*Gyps africanus*) in South Africa preferentially nest in larger trees, especially *Diospyros mespiliformis*, with tree size being a stronger predictor of nest presence than tree health. Although most nesting trees were healthy, vultures did not significantly avoid unhealthy trees, suggesting flexibility in health tolerance. Conservation efforts should focus on preserving large, structurally sound trees by managing elephant impact and mitigating habitat stress.

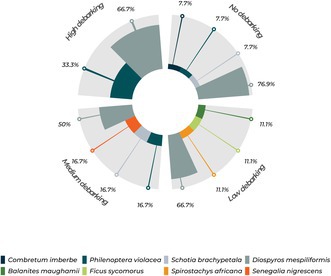

## 1 Introduction

Effective conservation strategies require comprehensive information on species' ecological requirements and the threats they face (Carter et al. 2019). Assessing these factors enables practitioners to design targeted management responses that protect species, their habitats, and the ecosystem services they provide. Wildlife monitoring is a fundamental part of this process, providing data on habitat conditions, and emerging threats (Stephenson 2019).

Globally, vultures face greater extinction risks than most other bird species (McClure et al. 2018). Most populations of vultures are undergoing severe declines (Prakash et al. 2012; Santangeli et al. 2019) particularly in Africa where experts have termed the situation “The African Vulture Crisis” (Ogada, Botha, and Shaw 2016). Two African vulture species—the Egyptian vulture ( *Neophron percnopterus* ) and the lappet‐faced vulture (*Torgos tracheliotos*) are categorized as Endangered, while the hooded vulture ( *Necrosyrtes monachus* ), Rüppell's vulture (*Gyps rueppelli*), white‐headed vulture ( *Trigonoceps occipitalis* ), and white‐backed vulture ( *Gyps africanus* ) are listed as Critically Endangered on the IUCN Red List (IUCN 2024; Ogada, Botha, and Shaw 2016). In African savannahs, vultures play a key role in controlling mesopredator populations, recycling nutrients, and likely helping to reduce the spread of pathogens (Den Heever et al. 2021). However, the specialized adaptations (soaring flight, social feeding etc.) that allow these obligate scavengers to efficiently find and dispose of carcasses (Kane et al. 2014; Den Heever et al. 2021) also predispose them to anthropogenic threats such as poisoning, collisions with wind turbines, and mortalities on powerlines (Ogada, Shaw, et al. 2016). Furthermore, while the anthropogenic threats to vultures are fairly well known (Ogada, Botha, and Shaw 2016), the natural threats are not as well studied. The latter can include infectious diseases, severe weather events, and impacts from non‐human species, such as predation, nest usurpation, and the loss of nesting trees through natural events such as fire, drought and elephant damage (Vogel et al. 2014; Rushworth et al. 2018). Indeed, increasing African savannah elephant ( *Loxodonta africana* ) populations in some areas and fire pressure may be intensifying the threats to breeding tree‐nesting vultures due to the associated damage and loss of their nesting trees (Walpole et al. 2004; Monadjem and Garcelon 2005).

One of Africa's most threatened vulture species is the White‐backed Vulture. This species has experienced a severe population decline, estimated at 63%–89% over the past three generations (BirdLife International 2024). White‐backed Vultures demonstrate preferences when selecting their nesting sites in African savannas. These birds favor tall, mature trees, with studies showing that average heights of nest trees range from over 7 m (Johnson and Murn 2022) to 17.2 m (Monadjem et al. 2016). In South Africa's Limpopo province, 15 out of 22 (68%) White‐backed Vulture nests along the Olifants River were in *Ficus sycomorus* (Monadjem et al. 2016). However, preferred nesting tree species will likely depend on which tree species have the favored traits and are available within the landscapes where White‐backed Vultures occur, over their vast distribution. The location of nesting trees follows distinct patterns in relation to landscape features. A recent study in our study area found that White‐backed Vultures tend to nest near water sources, while maintaining considerable distance from human disturbance (Hounnouvi et al. 2025). Nesting birds also had a strong affinity for woodland habitats, particularly those containing large, stable trees (Hounnouvi et al. 2025).

While previous studies across West, East, and Southern Africa have identified tree species preferences and general nesting habitat characteristics, fewer have focused explicitly on the role of tree health and natural disturbance in influencing vulture nest site selection. Moreover, habitat availability and nesting preferences can vary regionally depending on tree species composition, landscape structure, and the intensity of ecological pressures. This makes it critical to assess nesting ecology in diverse protected areas where conservation strategies must be tailored to local conditions. Among natural threats, elephant damage emerges as a particularly pressing concern. Multiple studies (Cook et al. 2025; Vogel et al. 2014) document the impact of elephant debarking on trees suitable for nesting, and vultures have been shown to actively avoid heavily damaged trees (Cook et al. 2025). Fire presents an additional threat to vulture nest trees (Virani et al. 2010), although its impact is less thoroughly explored.

To assess characteristics affecting nest tree selection in White‐backed Vultures, we chose a study area represented by Kempiana and Manyeleti Nature Reserves within the Greater Kruger National Park. We selected this area to explicitly examine elephant damage (e.g., bark stripping) as a potential factor affecting nest tree selection. Elephant numbers are increasing in the region (Ferreira et al. 2024; Louw et al. 2021), raising concern about the long‐term viability of suitable nesting trees. According to the Multi‐species Biodiversity Management Plan for Vultures in South Africa, the expanding elephant population in protected areas poses a growing threat to tree‐nesting vultures, underscoring the need for targeted research in key breeding sites such as our study area (DFFE 2024). Furthermore, vultures are species of special concern for South African National Parks (SANParks), which has overall management control of the Greater Kruger National Park, including our study area. Our study therefore addresses a critical gap in understanding the factors shaping White‐backed Vulture nest site selection under current ecological pressures. Specifically, we aimed to investigate the nesting habitat preferences of White‐backed Vultures in the Kempiana and Manyeleti Nature Reserves, with a focus on the structural characteristics and health conditions of nesting trees, as well as their potential interrelationships. We hypothesized that the species would prefer trees that are larger and in better health (with less damage to the trunk), as these features likely provide more stable and secure nesting platforms. The findings of this study will contribute to conservation strategies by identifying key habitat features essential for supporting breeding populations of this vulnerable species in the Greater Kruger landscape.

## 2 Methods

### 2.1 Study Area

**FIGURE 1:**
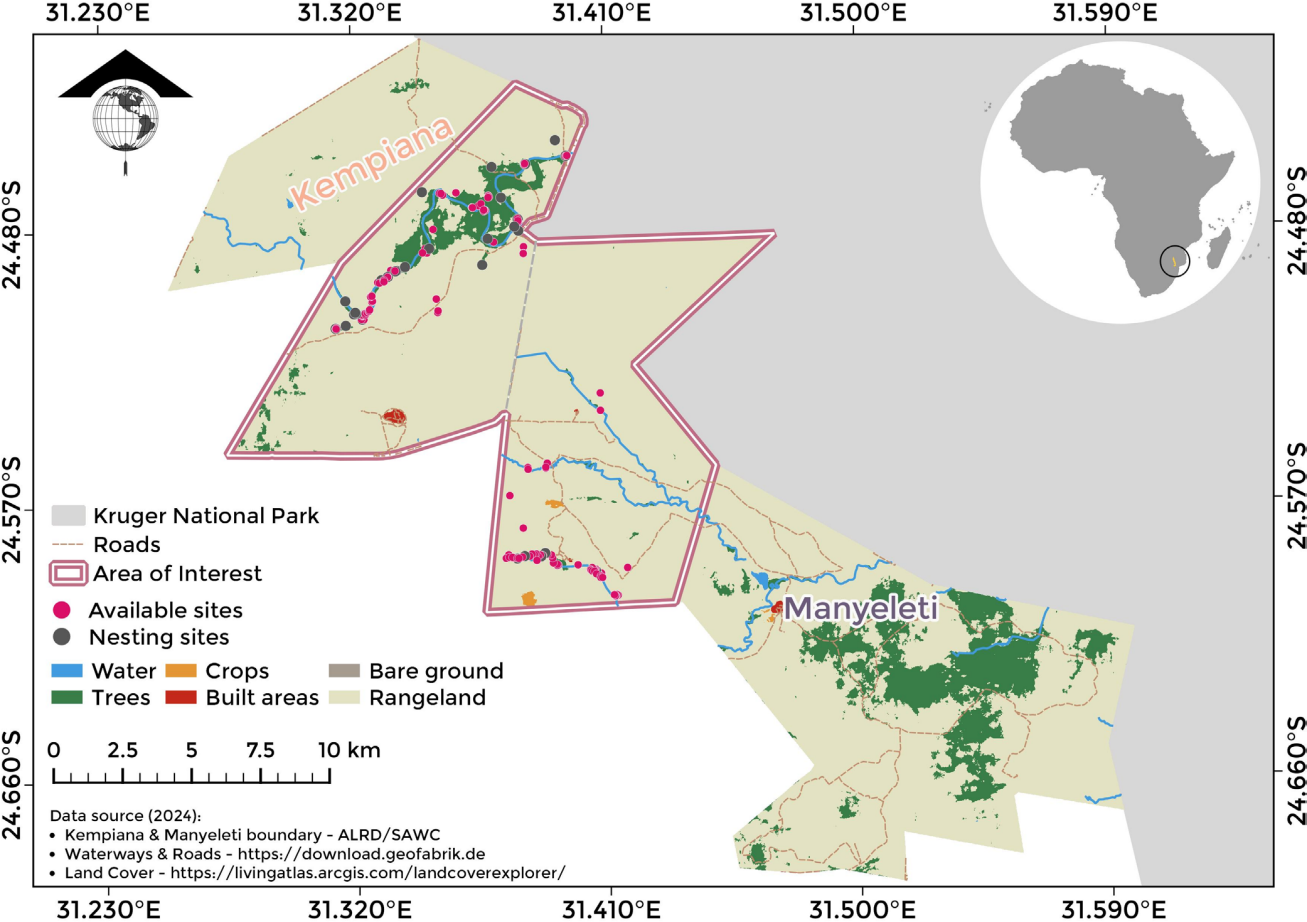
Study area (bounded by a pink, double line) in Kempiana and Manyeleti Nature Reserves (separated by gray dashed line), north‐eastern South Africa. White‐backed Vulture nesting trees are shown with gray dots (“nesting sites”, trees with nests), and randomly‐chosen trees are shown with pink dots (“available sites”, trees with no nests). For definitions of Land Use Land Cover (LULC) classes, see Table S1.

We conducted our study in Kempiana and Manyeleti Nature Reserves in north‐eastern South Africa (Figure 1). These two reserves, covering 368.77 km^2^, are open to one another and considered as one continuous landscape. Manyeleti Nature Reserve (31.473° E; −24.599° S), lies southwest of the Kruger National Park's (KNP) Orpen Gate. It borders Timbavati Private Nature Reserve and Sabi Sand Nature Reserve (Kozakowski 2024) and shares an unfenced 30 km eastern border with KNP (Briggs 2011). Initially consisting of several privately owned reserves divided by fences, these areas, along with adjacent reserves, came under the administration of Mpumalanga Province after 1994 (Marshal et al. 2012). The landscape in KNP and the surrounding areas is characterized by arid to semi‐arid wooded savannah, with a diverse mix of trees and grasses that vary spatially and temporally (du Toit et al. 2003). Across Kempiana and Manyeleti Nature Reserves, the dominant land cover is rangeland, comprising 89.67% of the area, followed by trees (9.92%), built area (0.14%), crops (0.12%), water (0.10%), and bare ground (0.05%) (see Supporting Information for class definitions).

### 2.2 Data acquisition and processing

The study was conducted in February and March 2024, during the non‐breeding season for White‐backed Vultures, prior to egg‐laying (Houston 1976; Johnson 2018). This timing ensured that the fieldwork did not disturb breeding vultures. Aerial imagery from the South African Environmental Observation Network (SAEON) (Wynand et al. 2024), was used to select survey locations across the study area, ensuring that the chosen sites were representative of suitable vulture nesting habitats. Previous studies have found that vultures often nest in taller trees (Chomba and M'Simuko 2013; Kemp et al. 2020), which are often located along rivers. We therefore selected sites along waterways and within open woodlands, where large trees are more likely to be found.

To identify potential nest trees, we surveyed trees that were at least 10 m tall and 1.4 m in circumference at 1.3 m above ground. These minimum measurements were based on previous studies of *G. africanus* nest tree sizes (e.g., Kendall et al. 2018). Trees considered for use by vultures are those with an indicator of active vulture presence, i.e., a nest present in the tree, fresh excreta and molted feathers under the trees (Postupalsky 1974; Zuberogoitia et al. 2016). For each nesting tree, we also recorded three control trees without nests in close proximity to the trees with nests (Vogel et al. 2014), within a 50 m radius, and with a minimum height of 10 m and a minimum trunk circumference (at 1.3 m above the ground) of 1.4 m. This ensured that we recorded a variety of tree parameters and habitat features, both for vulture nest trees and for randomly‐chosen (control) trees. The GPS coordinates of each tree were recorded, and we used a tape measure to measure tree trunk circumference at 1.3 m (CBH1) and at 0.3 m (CBH2) above ground level, as well as canopy width at the widest point of the canopy. To measure tree height H(m), we employed a clinometer‐based method. This involved measuring a horizontal distance d from the observer to the base of the tree using a measuring tape and then using a clinometer to record the angle θ of elevation from the observer's eye level to the top of the tree. With these two values we calculated H using basic trigonometric functions, specifically: $H=d*\tan\left(\theta \right)+h$, where h is the observer height at eye level (here 1.56 m).

Tree health was assessed by evaluating visible damage to the trunk, including fire damage, insect infestation, fungal presence, and debarking by elephants. Each type of damage was assigned a severity level from 0 to 3 based on the affected area: 0 (no damage, < 5% of the tree affected), 1 (5%–25% of the tree affected), 2 (25%–50% affected), and 3 (> 50% affected). This method was adapted from Vogel et al. (2014), who recorded impact intensity on a scale of 10.

We processed the data in R (R Core Team 2023), performing transformations with the *dplyr* package (Wickham et al. 2024). The trees were classified as “Unhealthy” if one damage type was equal to, or exceeded, the median of the severity levels (1.5 in this study, i.e., median of {0, 1, 2, 3}) and “Very unhealthy” if multiple damage types exceeded this median; otherwise, trees were classified as “Healthy” (equation E). Finally, we derived the scientific names of trees using a field guide (van Wyk 2013).

$$\left(E\right)\;H_{s}=\left\{\begin{array}{c} \text{Healthy}\;\text{if}\;\sum_{i=1}^{n}I\left(d_{i}\geq 1.5\right)=0\\ \text{Unhealthy}\;\text{if}\;\sum_{i=1}^{n}I\left(d_{i}\geq 1.5\right)=1\\ \text{Very unhealthy}\;\text{if}\;\sum_{i=1}^{n}I\left(d_{i}\geq 1.5\right)>1\; \end{array}\right.$$

where $H_{s}$ is health status, n is number of severity levels (four in this study), $d_{i}$ is severity levels for each type of damage on a tree (e.g., {2, 0, 1, 0} for debarking, fire, insect and fungus respectively), $I\left(d_{i}\geq 1.5\right)$ is an indicator function that returns 1 if $d_{i}\geq 1.5$ and 0 otherwise.

### 2.3 Data Analysis

We performed our analyses on both the White‐backed Vulture nest trees (*n* = 31) and randomly chosen (control) trees (*n* = 174) starting by investigating the potential association between tree health status and various tree size measurements using Kruskal‐Wallis tests to determine whether there were significant differences in tree health status across different tree sizes. We then built a binomial logistic regression model to assess the influence of tree size measurements (tree height, canopy width and circumference) and health status on vulture nest presence. Prior to building the generalized linear model (GLM), we assessed multicollinearity among the predictor variables using the Variance Inflation Factor (VIF) approach, implemented via the *usdm* R package (Naimi et al. 2014). The VIF results indicated that none of the variables exhibited problematic multicollinearity, as all VIF values (ranging from 1.18 to 1.88) were well below the commonly used threshold of 5. The highest pairwise correlation among the predictors was 0.61 (between canopy width and CBH2), which is considered moderate and acceptable. Based on these results, all variables were retained for inclusion in the model. All data visualizations were made using the *ggplot2* R package (Wickham 2016), and the map of the study area was created with QGIS (v.30.28.11).

## 3 Results

**FIGURE 2:**
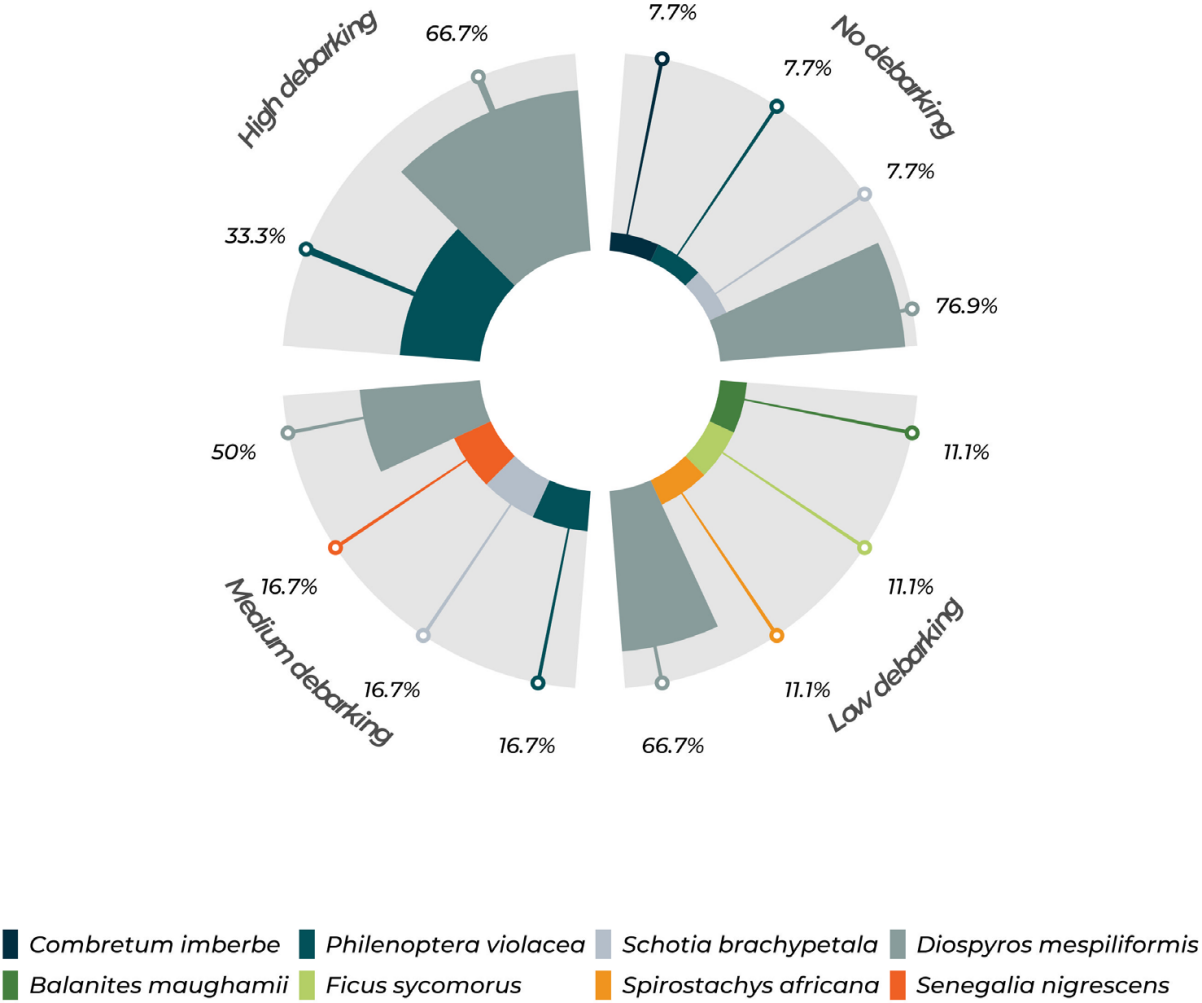
Distribution of tree species hosting White‐backed Vulture nests (*n* = 31) across elephant debarking intensity categories.

We obtained measurements on 205 individual trees within the area of interest, with 19 different species represented. Vulture nests were found primarily in trees (*n* = 16, 51.6%) and rangeland (*n* = 15, 48.4%), whereas available (non‐nesting) sites were also dominated by rangeland (*n* = 103, 59.1%) followed by trees (*n* = 71, 40.9%), indicating a relatively balanced use of both vegetation types for nesting despite rangeland being more widely available. The most common tree species was *Diospyros mespiliformis* , with 47.3% of the trees surveyed. Other common tree species included *Combretum imberbe* (9.8%), *Sclerocarya birrea* (6.8%), and *Philenoptera violacea* (6.8%). Most (74.2%) of our sample of 205 trees were healthy, 18.1% were unhealthy, and 7.8% were very unhealthy. The tree species most affected by damage included *Senegalia nigrescens*, *Xanthocercis zambesiaca*, and *Diospyros mespiliformis* (Figures S1 and S2) These trees were notably tall, with *X. zambesiaca* averaging 12.2 m in height (95% CI: 9.7–14.7 m), *D. mespiliformis* at 14.9 m (95% CI: 14.2–15.6 m), and *S. nigrescens* at 14.1 m (95% CI: 12.4–15.7 m). Figure 2 shows the distribution of tree species supporting active vulture nests across four categories of debarking intensity. *Diospyros mespiliformis* emerged as a highly prominent species, consistently appearing across all debarking levels, accounting for 76.9% of trees with no debarking, 66.7% of those with low debarking, 50% of trees with medium debarking, and 66.7% of trees with high debarking. *Philadenoptera violacea* was found in both no debarking (7.7%) and high debarking (33.3%).

**FIGURE 3:**
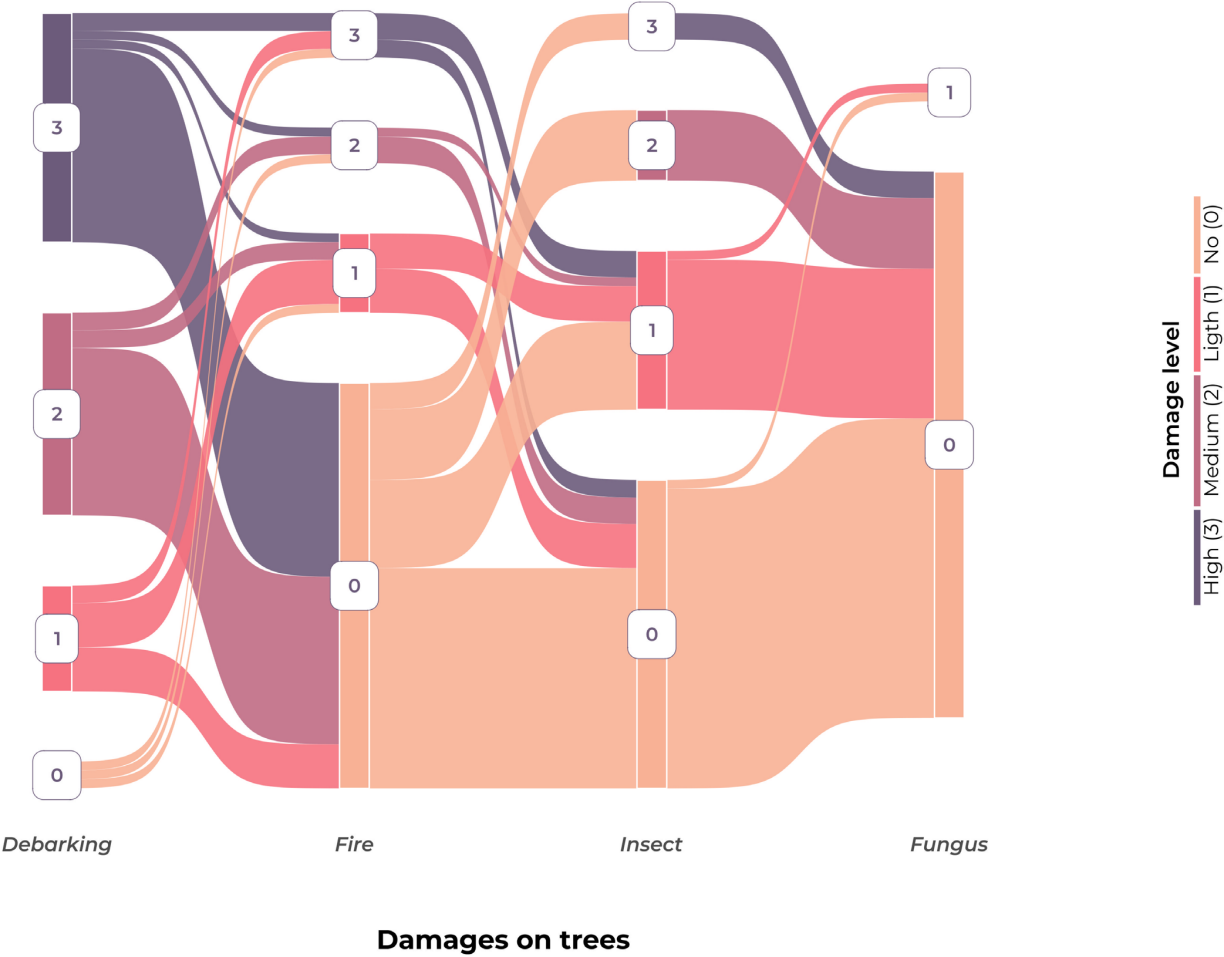
Interconnectivity between different types of tree damage and their respective severity. The width of nodes in the diagram is proportional to the number of trees affected at each severity level connected to the severity in another damage, following the flows from left to right.

Debarking was the most frequent type of damage, and the most severe (Figure 3). Many of the trees with high debarking (*n* = 26, 12.7.%) had no fire damage. Only a few trees had low (*n* = 9, 4.4%) or high‐severity (*n* = 5, 2.5%) fire damage. Trees with no insect damage (*n* = 173, 84.4%) were more frequent than trees with light (*n* = 21, 10.2%), medium (*n* = 8, 3.9%) or high‐severity (*n* = 3, 1.5%) insect damage. We found that the trees with some insect damage (light, medium or high) were often debarked, regardless of debarking severity. The occurrence of insect damage can thus be linked to tree debarking, confirmed by a Fisher's exact test (*p* < 0.001). Fungus damage was less common than the other types of damage among our sample of trees; among the unhealthy trees, just two had fungus.

**FIGURE 4:**
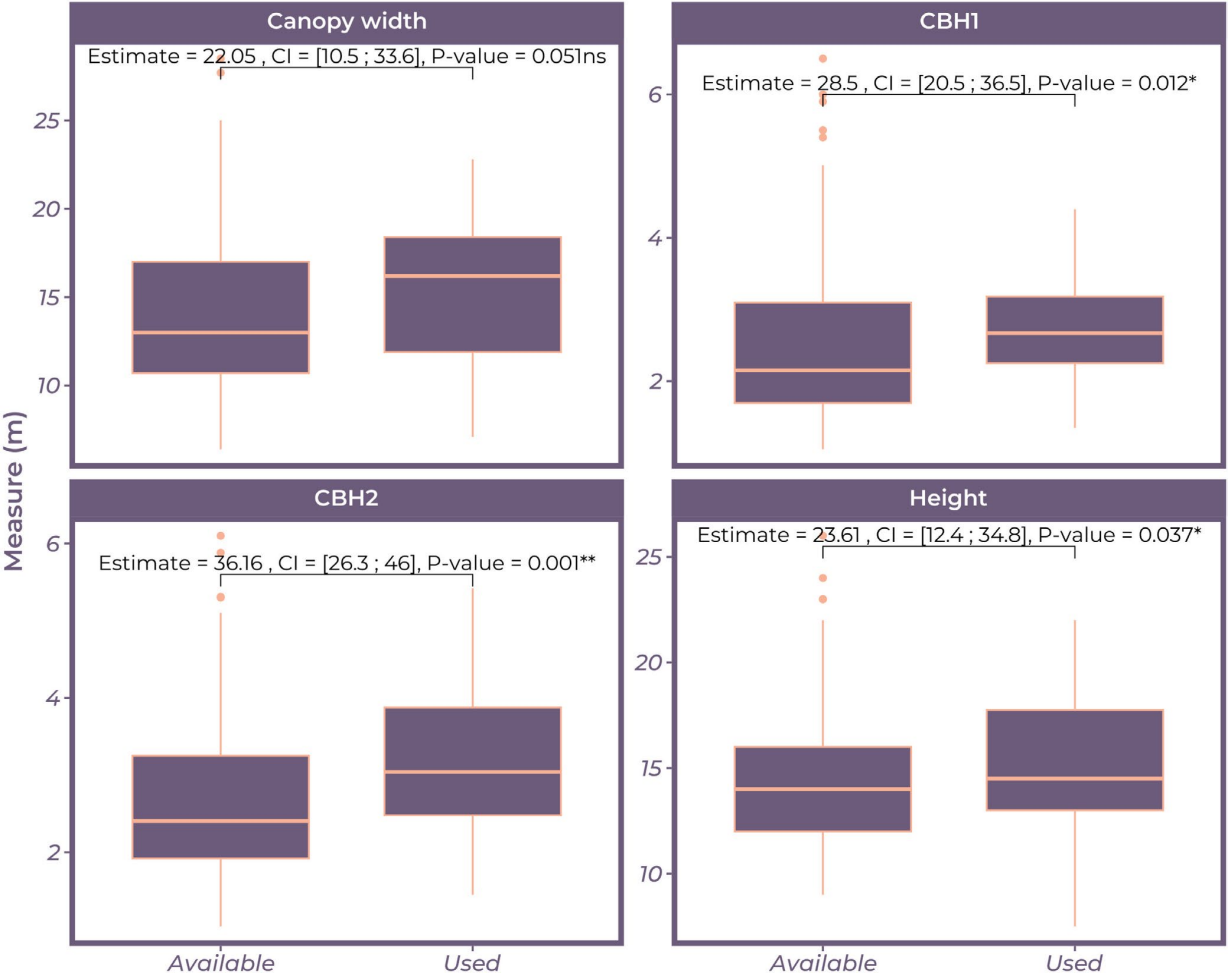
Post‐hoc Kruskal‐Wallis test for association between vulture presence and tree measures. “No Nest” represents trees with no nest presence in their branches, and “Nest” is the opposite. Asterisk “*”. means significant difference and “ns” otherwise. CI is 95% Confidence Interval.

We investigated the potential association between tree health status and various tree measurements (height, circumferences, and canopy width), but none of the tested measures showed a significant association with tree health status. Specifically, the Kruskal‐Wallis test results indicated no substantial differences in tree health levels based on tree height (statistic = 3.367, *p* = 0.186), CBH1 (statistic = 0.106, *p* = 0.948), CBH2 (statistic = 0.233, *p* = 0.890), or canopy width (statistic = 0.647, *p* = 0.724), as all *p*‐values were > 0.05. For CBH1, CBH2 and height, the values for vulture nest trees and control trees were significantly different, indicating that trees with vulture nests had significantly larger CBH1, CBH2 and height than trees without vulture nests (Figure 4).

The logistic regression analysis revealed that CBH2 was a significant predictor (Table 1) of vulture nest presence (*z* = 2.175, *p* = 0.030), with an estimated coefficient of 0.587 ± 0.270. This positive coefficient suggests that for each unit increase in CBH2, the odds of a tree being selected by White‐backed Vultures for nesting increase.

**TABLE 1:** Generalized Linear Model (GLM) examining the influence of tree characteristics on the probability of White‐backed Vulture ( *Gyps africanus* ) nesting presence.

Predictor	Estimate	Standard error	*Z*	*p*	Significance
CBH2	0.587	0.270	2.175	0.030	*
CBH1	−0.125	0.269	−0.464	0.643	ns
Height	0.085	0.062	1.381	0.167	ns
Canopy width	−0.020	0.061	−0.336	0.737	ns
Health level—unhealthy	0.698	0.496	1.407	0.159	ns
Health level—very unhealthy	−0.198	0.807	−0.245	0.806	ns

*Note:* Significant predictors (*p* value < 0.05) are marked with an asterisk.

Abbreviation: ns, non‐significant.

## 4 Discussion

We found that Critically Endangered White‐backed Vultures seem to select nest trees based on their size (particularly CBH and height), but not on the trees' health status. *Diospyros mespiliformis* was the tree species most commonly selected for White‐backed Vulture nesting in our study area, which contrasts with the results of Kemp and Kemp (1975) in the Kruger National Park, who found that *Senegalia nigrescens* was the main nesting tree. The contrast is echoed in more recent research by Cook et al. (2025), where *S. nigrescens* accounted for most nests (55.6%), while *D. mespiliformis* comprised a smaller proportion (14.3%) as the second potential nesting tree. This change of preferred nest tree species within 50 years could be explained by the fact that *S. nigrescens* is among the tree species most often damaged by elephants and fire (Cook et al. 2025; Shannon et al. 2011), ultimately becoming unavailable for nesting sites in our study area. Elephants preferentially debark trees characterized by strong and pliable bark rich in fibrous tissue—traits that make species like *Senegalia nigrescens* and *Sclerocarya birrea* easier to strip and thus more susceptible to damage (Malan and van Wyk 1993). Additionally, the discrepancy in nesting tree preferences across studies may reflect regional differences in tree species availability, habitat structure, or varying levels of elephant impact. *D. mespiliformis* , often found in riparian areas less heavily impacted by elephants, may offer more stable and longer‐lasting nesting opportunities. Alternatively, the difference may be because we focused our fieldwork along waterways, and *Diospyros mespiliformis* occurs in riparian habitat. According to several studies across Africa, *G. africanus* select several tree species for nesting; for example, in Ghana, the cotton tree *(Ceiba pentandra)* is selected for nesting (Deikumah 2020), in addition to other tree species such as *Faidherbia albida* , and *Vachellia xanthophloea* (Chomba and M'Simuko 2013; Moradiya and Goswami 2021). Large trees are important for *G. africanus* because they need to build substantial nests that can hold their own weight (both adults) – up to 5.4 kg each, and the weight of a fledgling (Chomba and M'Simuko 2013; Kemp et al. 2020). These differences highlight the importance of considering local habitat conditions and elephant densities when assessing nesting site availability and planning conservation actions for vulture populations.

Our study found that unhealthy trees, particularly those affected by debarking, did not influence vulture nesting sites. Elephant damage through debarking did not impact nest persistence, even over a 5‐year period (Vogel et al. 2014). This may reflect the tendency of vultures in our study area to nest in large *D. mespiliformis* trees, which typically exceed the vulnerable 5–8 m height class most susceptible to elephant damage (Turner et al. 2022; Vogel et al. 2014). Due to strong site fidelity, vultures may continue using damaged trees for several years before these trees ultimately collapse. This lag between damage and tree mortality highlights the need for long‐term monitoring of nest trees to understand how damage accumulates and affects nest and nest tree persistence over time. Although we did not have data on tree age, future research should aim to assess how tree age relates to susceptibility to different types of damage and to determine thresholds at which trees become unsuitable for nesting. Cook et al. (2025) similarly emphasized the importance of tracking treefall and regeneration rates to predict when vultures may face shortages of suitable nest trees. Incorporating such long‐term, individual‐based monitoring into management strategies will be essential for anticipating habitat loss and sustaining breeding populations in elephant‐occupied landscapes. In the Associated Private Nature Reserves (APNR) which border the Kruger National Park, *S. nigrescens* and *D. mespiliformis* hold the majority of nests, but elephants debark *S. nigrescens* and *Sclerocarya birrea* heavily while largely sparing riverine *D. mespiliformis* and *Philenoptera violacea* (Cook and Henley 2019). Our results align with these results, and so we recommend a focus on *D. mespiliformis* for vulture conservation efforts due to its consistent and significant use by nesting White‐backed Vultures in our study area across various debarking levels. Additionally, the presence of insects and fungi on large trees was negatively associated with tree survival, suggesting that elephant‐induced damage may indirectly facilitate insect and fungal attacks, ultimately reducing a nest tree's lifespan. Trees that were healthy, unhealthy, or very unhealthy were not characterized by any tree measure. In other words, the damage observed (in terms of fires, insects, or elephant damage) can affect any size of tree.

Bark removal increases fire‐induced xylem damage and this damage contributes to stem mortality (Moncrieff et al. 2008). In our study, the link between damage types is more noticeable with debarking and insect damage than with debarking and fire. This difference could be explained by the fact that we had only a few trees that were damaged by fire in our sample, and fire was a less noticeable threat than debarking on the surveyed trees. The position of these large trees along riverbanks means there is more soil moisture which enables faster tree growth and keeps the undergrowth moist for longer, thereby inhibiting hot fires that destroy trees. One of the limitations of this study is that our study area most likely has higher densities of elephants than other locations across Africa where White‐backed Vultures breed, and so the levels of elephant damage to nest trees in our study area may not be representative of the levels in other areas of Africa where this vulture species breeds (e.g., in South Africa's Kalahari area, there are no elephants).

The fungi on our surveyed trees are most likely saprophytic, as they were growing on the sides of the trees, which were likely damaged. While polypore mushrooms can eventually weaken a tree, their presence is not necessarily a cause for concern in healthy trees (Kobza et al. 2022). Further conclusions on the impact of these mushrooms on *S. brachypetala* are beyond the scope of this study.

We have shown that along the waterways of Manyeleti and Kempiana Nature Reserves, *Diospyros mespiliformis* was the tree species that was most selected for nesting by White‐backed Vultures. We noted numerous “unhealthy” vulture nest trees and control trees with damage. The debarking by elephants was the most severe and common type of damage to these trees. Vulture nest presence did not depend on tree health status; instead vultures appeared to select nesting trees based on their size. This study provides a basis for allowing us to better understand how White‐backed Vultures select their nest trees, in turn providing conservation practitioners with information on which trees they should protect to aid in the conservation of this Critically Endangered vulture species in north‐east South Africa.

## Author Contributions

**Stanislas Mahussi Gandaho:** conceptualization (equal), data curation (equal), formal analysis (equal), investigation (equal), methodology (equal), validation (equal), writing – original draft (equal), writing – review and editing (equal). **Ezéchiel Fidèle Koffi Hounnouvi:** data curation (equal). **Lindy Jane Thompson:** conceptualization (equal), methodology (equal), supervision (equal), writing – review and editing (equal). **Fern Bain:** data curation (equal). **Paul Scholte:** writing – original draft (equal). **Peter Hamming:** writing – original draft (equal).

## Conflicts of Interest

The authors declare no conflicts of interest.

## Supporting information


**Appendix S1:** ece372545‐sup‐0001‐AppendixS1.docx.

## Data Availability

The data and R code that support the findings of this study are openly available (Gandaho et al. 2025). https://zenodo.org/records/17618013.
