# Correction to “The Influence of Tree Characteristics on White‐Backed Vulture (*Gyps africanus*) Nest‐Site Selection in Manyeleti and Kempiana Nature Reserves, South Africa”

**DOI:** 10.1002/ece3.72632

**Published:** 2025-12-10

Gandaho, S.M., Hounnouvi, E.F.K., Thompson, L.J., Bain, F., Scholte, P. and Hamming, P. (2025), The Influence of Tree Characteristics on White‐Backed Vulture (*Gyps africanus*) Nest‐Site Selection in Manyeleti and Kempiana Nature Reserves, South Africa. Ecol Evol, 15: e72545. https://doi.org/10.1002/ece3.72545.

The country for the second affiliation should be listed as the Democratic Republic of the Congo, not the Republic of the Congo. The correct affiliation is as follows:

“ERAIFT, University of Kinshasa, Kinshasa, Democratic Republic of the Congo.”

The online version of this article has been corrected accordingly.

We apologize for this error.

