# Supplementary material for: The Influence of Tree Characteristics on White‐Backed Vulture (Gyps africanus) Nest‐Site Selection in Manyeleti and Kempiana Nature Reserves, South Africa
Source: Ecol Evol. 2025 Nov 17;15(11):e72545. doi: 10.1002/ece3.72545 (PMC12623063; doi:10.1002/ece3.72545)
Supplement: Supplementary file 1 — Appendix S1: ece372545‐sup‐0001‐AppendixS1.docx. [file ECE3-15-e72545-s001.docx]

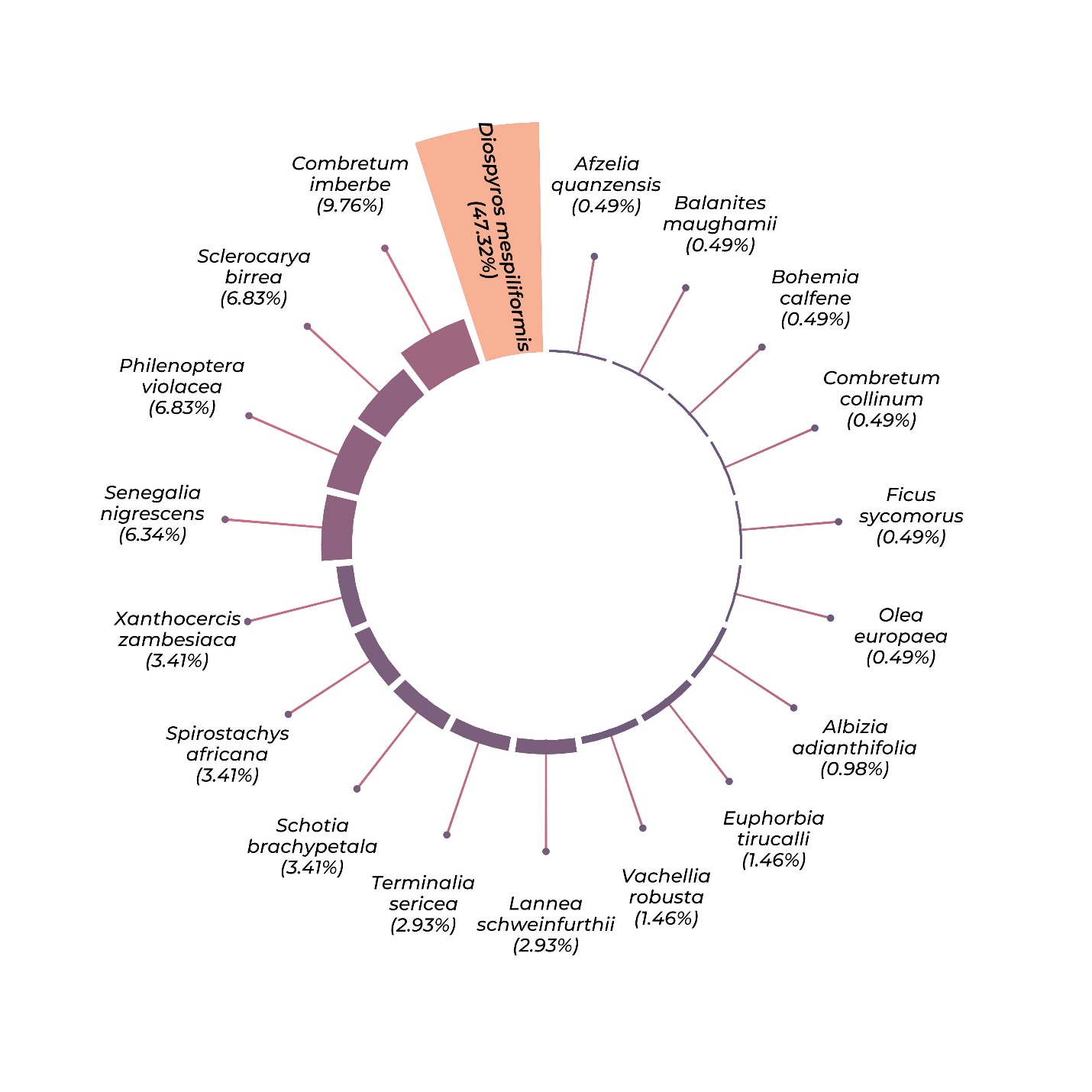


Figure S1. The diversity of tree species in which White-backed Vulture nests were recorded during surveys conducted in Kempiana and Manyeleti Nature Reserves, South Africa, in 2024 (N = 205).


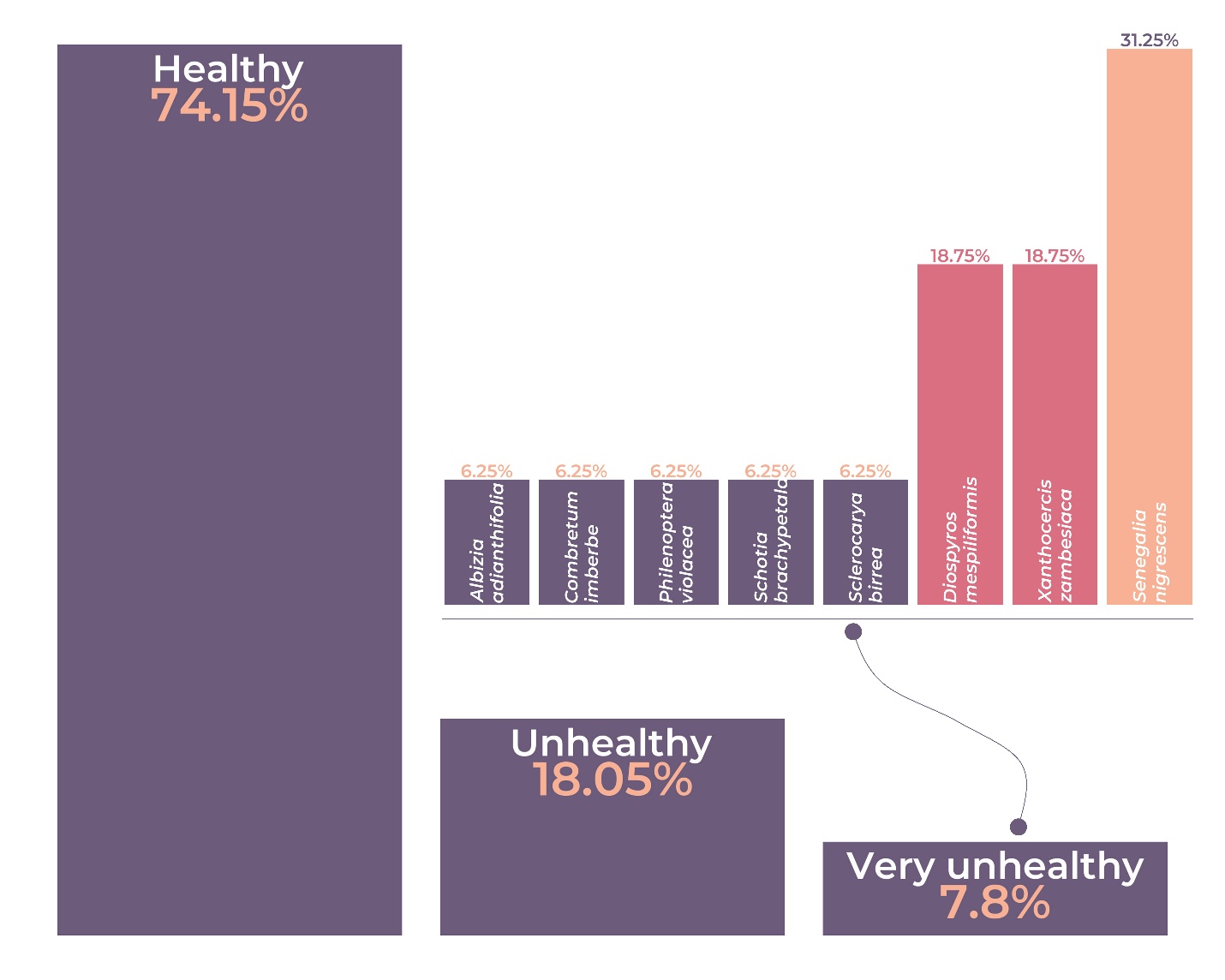


Figure S2: Health distribution of trees surveyed in Kempiana and Manyeleti Nature Reserves, South Africa (N = 205). All trees had a circumference (at 1.3m above the ground) of ≥1.4m, and a height of ≥10m.

Table S1: Land Use Land Cover (LULC) class definitions

| Name | Description |
| --- | --- |
| Water | Areas where water was predominantly present throughout the year; may not cover areas with sporadic or ephemeral water; contains little to no sparse vegetation, no rock outcrop nor built up features like docks; examples: rivers, ponds, lakes, oceans, flooded salt plains. |
| Trees | Any significant clustering of tall (~15 feet or higher) dense vegetation, typically with a closed or dense canopy; examples: wooded vegetation, clusters of dense tall vegetation within savannas, plantations, swamp or mangroves (dense/tall vegetation with ephemeral water or canopy too thick to detect water underneath). |
| Flooded vegetation | Areas of any type of vegetation with obvious intermixing of water throughout the majority of the year; seasonally flooded area that is a mix of grass/shrub/trees/bare ground; examples: flooded mangroves, emergent vegetation, rice paddies and other heavily irrigated and inundated agriculture. |
| Crops | Human planted/plotted cereals, grasses, and crops not at tree height; examples: corn, wheat, soy, fallow plots of structured land. |
| Built Area | Human made structures; major road and rail networks; large homogenous impervious surfaces including parking structures, office buildings and residential housing; examples: houses, dense villages / towns / cities, paved roads, asphalt. |
| Bare ground | Areas of rock or soil with very sparse to no vegetation for the entire year; large areas of sand and deserts with no to little vegetation; examples: exposed rock or soil, desert and sand dunes, dry salt flats/pans, dried lake beds, mines. |
| Snow/Ice | Large homogenous areas of permanent snow or ice, typically only in mountain areas or highest latitudes; examples: glaciers, permanent snowpack, snow fields. |
| Clouds | No land cover information due to persistent cloud cover. |
| Rangeland | Open areas covered in homogenous grasses with little to no taller vegetation; wild cereals and grasses with no obvious human plotting (i.e., not a plotted field); examples: natural meadows and fields with sparse to no tree cover, open savanna with few to no trees, parks/golf courses/lawns, pastures. Mix of small clusters of plants or single plants dispersed on a landscape that shows exposed soil or rock; scrub-filled clearings within dense forests that are clearly not taller than trees; examples: moderate to sparse cover of bushes, shrubs and tufts of grass, savannas with very sparse grasses, trees or other plants. |
